# The TOR Signaling Pathway Governs Fungal Development, Virulence and Ustiloxin Biosynthesis in *Ustilaginoidea virens*

**DOI:** 10.3390/jof11040239

**Published:** 2025-03-21

**Authors:** Yuejiao Li, Shuqin Sun, Guangsheng Li, Zezhong Yang, Yuqi Xing, Ruixiang Wang, Yuanhu Xuan, Xiurong Yang

**Affiliations:** 1 Institute of Plant Protection, Tianjin Academy of Agricultural Sciences, Tianjin 300381, China; molly_lyj@126.com (Y.L.); sunshuqin688@126.com (S.S.);; 2 State Key Laboratory of Elemento-Organic Chemistry and Department of Plant Protection, National Pesticide Engineering Research Center (Tianjin), Nankai University, Tianjin 300071, China

**Keywords:** *Ustilaginoidea virens*, target of rapamycin (TOR) pathway, mycelial growth, pathogenicity, mycotoxins

## Abstract

*Ustilaginoidea virens* is an economically important plant pathogen that causes rice false smut, which causes yield reduction and produces mycotoxins in infected grains that pose a serious threat to human and animal health. The target of rapamycin (TOR) signaling pathway acts as a master regular in regulating cell growth and secondary metabolism in fungi. However, little is known about the function of the TOR pathway in regulating fungal development, pathogenicity and mycotoxin biosynthesis in *U. virens*. Here, we demonstrate that the TOR signaling pathway positively regulates the cell growth, conidiation and pathogenicity in *U. virens* through the biochemical inhibition of TOR kinases. The inhibition of TOR in *U. virens* (*UvTOR*) by rapamycin significantly induces the expression of genes related to mycotoxin biosynthesis, especially that of ustiloxins. Transcriptome analysis under TOR inhibition revealed that the TOR signaling pathway is a regulatory hub that governs *U. virens* growth and metabolism. A total of 275 differentially expressed genes (DEGs), consisting of 109 up-regulated DEGs and 166 down-regulated DEGs, were identified after rapamycin treatment. The up-regulated DEGs were enriched in amino acid- and acetyl-CoA-related metabolism pathways and the down-regulated DEGs were enriched in carbohydrate- and fatty acid-related metabolism pathways. Collectively, our results provide the first in-depth insight into the TOR signaling pathway in regulating vegetable growth, virulence and mycotoxin biosynthesis in *U. virens*.

## 1. Introduction

Rice false smut (RFS) is caused by the fungal pathogen *Ustilaginoidea virens*, which infects rice stamen filaments and colonizes surrounding rice florets to form false smut balls that are several times larger than the grain [1,2]. With the wide cultivation of semi-dwarf high-yielding rice varieties, the adoption of higher crop density planting methods and the increasing application of nitrogen fertilizer, the occurrence of RFS has seen a sharp increase, causing a yield loss of 20–30% each year and even up to 70% in severe cases [3]. *U. virens* not only causes a significant reduction in grain yield but also seriously threatens rice quality by producing multiple types of mycotoxins which exhibit toxic effects on plants, animals and humans [4]. As the predominant type of mycotoxins only found in rice false smut balls generated by *U. virens*, the water-soluble cyclopeptide ustiloxins have seven derivatives, in which ustiloxin A is the main component [5]. The ustiloxin synthesis gene cluster, containing at least thirteen genes, has been predicted to be responsible for ustiloxin biosynthesis and modification in *U. virens* [6,7]. Ustiloxins exhibit a variety of biological activities, including inhibitory effects on microtubule assembly and cell skeleton formation in plants and animals [8,9,10]. In addition, ustiloxins might gradually accumulate in rice and become a potential health risk in humans; the detection rate of ustiloxin A was 82.1% among 312 rice samples worldwide [11,12]. Therefore, it is necessary to identify the regulatory mechanism of ustiloxin biosynthesis in *U. virens*.

The false smut balls are generated through hijacking the nutrient reservoir of rice spikelets and transferring nutrients to *U. virens* [13,14]. The coordinate control of nutrient metabolism and plant immunity suppression facilitate the infection of *U. virens.* In eukaryotic cells, the target of rapamycin (TOR) pathway responds to nutrients and growth factors to regulate vegetable growth and diverse metabolic pathways [15]. Rapamycin binds to the intracellular rapamycin receptor, the peptidyl-prolyl isomerase Fkbp12 (FK506 binding protein 12 kDa), and forms a complex that then directly binds and inhibits TOR kinases. The TOR proteins contain five functional domains, including multiple HEAT repeats (Huntingtin, elongation factor 3 (EF3), a subunit of protein phosphatase 2A (PP2A) and TOR1), a FAT domain (FRAP, ATM and TRRAP), a FRB (FKBP12–rapamycin-binding) domain, a kinase and a FATC (carboxy-terminal FAT) domain, which reside in the TOR protein between the N-terminal and the C-terminal [16,17]. Most fungi, including *Fusarium graminearum* [18], *Aspergillus flavus* [19] and *Magnaporthe oryzae* [20], have a single TOR kinase. However, some yeast species, such as *Saccharomyces cerevisiae* and *Schizosaccharomyces pombe*, possess two TOR orthologs [21]. In *S. cerevisiae*, Tor1 interacts with Tor2, Kog1, Lst8 and Tco89 to form TOR complex 1 (TORC1), which is sensitive to rapamycin, responds to nutrients and regulates cell growth by controlling ribosome biogenesis, protein synthesis and transcription [22,23]. TORC2, which is insensitive to rapamycin, recruits Lst8, Avo1, Avo2, Avo3, Bit61 and Bit2 to regulate the organization of the actin cytoskeleton [22].

Multiple studies have indicated that TOR is a master regulator of growth and metabolism of yeast, mammals and plants. Nonetheless, few reports focus on the TOR signaling pathway in filamentous fungi. The antifungal activity of rapamycin against phytopathogenic fungi, such as *F. graminearum* [18], *Aspergillus* species [19,24], *M. oryzae* [20], *B. cinerea* [25], suggested the role of the TOR pathway is similar in plant pathogens. In *F. graminearum*, it was reported that the TOR pathway regulated mycelia growth, conidiation, virulence and DON toxin production through phosphorylating downstream kinase FgSch9 [26]. In addition, the results in *M. oryzae* elucidated that MoTor is a powerful inhibitor that acts on the cAMP/cPKA signaling pathway to control appressorium formation and further influence virulence [20]. Currently, the physiological function of TOR in *U. virens* is unknown.

In this study, we systematically characterized the regulation of the TOR signaling pathway in vegetable growth, conidiation and virulence in *U. virens*. RNA-Seq analyses revealed that UvTOR negatively regulates the expression of genes related to amino acid and acetyl-CoA metabolism pathways and positively regulates the expression of genes related to carbohydrate and fatty acid metabolism pathways. We also showed that TOR affected the expression of genes involved in mycotoxin biosynthesis, especially that of ustiloxins. Our results suggest that UvTOR functions as a central regulator of vegetative growth, secondary metabolisms and pathogenicity and can act as a potentially efficient target in the control of rice false smut.

## 2. Materials and Methods

### 2.1. Plant Materials and Fungal Strains

*Ustilaginoidea virens* strain 57-2 was used as the wild-type (WT) strain. The fungal strain was grown on potato sucrose agar (PSA) medium (200 g/L potato, 20 g/L sucrose and 15 g/L agar) at 28 °C and mycelium disks were placed in potato sucrose (PS) liquid medium on a shaker at 150 rpm at 28 °C for 5 days to obtain conidia.

The susceptible *Oryza sativa* L. spp indica cultivar “Chunyou919 (CY919)” was used in this experiment. The plants were grown in the netting house at Wuqing Experimental Base in Tianjin (39.45′ N, 116.92′ E), with a row pitch and space of 20 cm.

### 2.2. Bioinformatics Analysis

TOR complex gene homologues were predicted in the *U. virens* genome through Pfam and NCBI BLAST (version 2.16.0) analyses. The phylogenetic tree was constructed using MEGA software (version 5.05) with the Neighbor-Joining method (1000 bootstraps). The TOR protein sequences of *Aspergillus nidulans* (XP_050467029.1), *Penicillium chrysogenum* (XP_056563482.1), *Fusarium graminearum* (XP_011320682.1), *U. virens* (XP_042994308.1), *Ustilago maydis* (XP_011389648.1) and *Saccharomyces cerevisiae* (NP_012600.1 and NP_012719.2) were downloaded from NCBI (https://www.ncbi.nlm.nih.gov/, accessed on 9 May 2023). The conserved amino acid residues in the catalytic motifs in different fungal species were identify using the MEME suite (version 5.5.7) (https://meme-suite.org/meme/tools/meme, accessed on 9 May 2023).

### 2.3. Microscopic Examinations of Hyphae and Conidiation

For the growth inhibition assay, 1 mg/mL of original liquid of rapamycin were used with ethyl alcohol as solvent. Then, 5 mm-diameter agar plugs with fungal mycelium were placed on PSA plates amended with rapamycin at 0, 2.5, 10 or 25 ng/mL, such that the final volume of ethyl alcohol of each plate was 0.25 μL. The diameter of the colony was recorded and measures after being cultured at 28 °C for 14 days. Each treatment consisted of three replicates. The hyphal morphology of each treatment was observed with a Nikon ECLIPSE NI-U research microscope (Nikon, Tokyo, Japan) using fresh mycelia harvested from colonies after growing on PSA plates for 14 days.

For the conidia production assay, five mycelial plugs of the WT stain were inoculated in 50 mL of PS liquid medium supplemented with rapamycin at 0, 25, 250 or 500 ng/mL. After incubation in a shaker with 150 rpm at 28 °C for 5 days, spores were collected by centrifugation and counted by a hemocytometer.

### 2.4. Infection Assay of U. virens

The inoculation protocol was conducted as described by Liu with minor modifications [27]. Briefly, the WT strain amended with rapamycin at 10 ng/mL was cultured in PS liquid medium in a shaker at 150 rpm at 28 °C for 5-7 days. The conidial suspension, with a concentration of 2 × 10^6^, was injected into panicles of the susceptible cultivar CY919 at about 5 days before rice booting. The controls were injected with PS liquid supplement with ethyl alcohol. After inoculation, all rice plants were covered with black sun-shading net and water was automatically sprayed every 12 h for 10 min to maintain a high humidity during the first three days after inoculation. At least 30 rice panicles were inoculated for each treatment. The numbers of diseased spikelets and rice smut balls per panicle were counted and recorded at 6 weeks post-inoculation. Disease incidence was recorded as the number of diseased plants relative to the total number of plants assessed [28,29]. This assay was independently repeated three times.

### 2.5. Quantification of Ustilaginoidins by HPLC

The quantitative examination of ustilaginoidins by HPLC was performed according to our previous published articles [4]. *U. virens* strain 57-2 was cultured on PSA plates amended with 10 ng/mL rapamycin or ethyl alcohol (as solvent control) at 28 °C for 28 days. The mycelia (1g) were collected and immersed in 50 mL EtOAc for 24 h. The extracts were dried and dissolved in methanol. Samples (10 μL) were loaded on an Agilent ZORBAX SB-C18 column (5 μm, 150 × 4.6 mm; Agilent Technologies, Polo Alto, CA, USA) and then eluted using an Agilent 1290 Infinity UHPLC system (Agilent, Waldbronn, Germany) at 30 °C with a UV detector (wavelength 290 nm) at the flow rate of 1 mL/min. The extracts were eluted with methanol using the following gradient: 0–2 min, 50% methanol; 2–12 min, 50–70% methanol; 12–27 min, 70–90% methanol; 27–52 min, increased to 99% methanol; and 52–55 min, 100% methanol.

### 2.6. Transcriptome Sequencing and Analysis

The hyphae of five plates of *U. virens* grown on PSA medium amended with 10 ng/mL rapamycin or ethyl alcohol (as solvent control) for 14 d at 28 °C were collected and mixed for RNA extraction. For each treatment, three independent biological replications at intervals of 3 days were performed. Total RNAs were extracted using a TRIzol reagent kit (Invitrogen, Carlsbad, CA, USA) and were subject to quality control and quantification using an Agilent 2100 Bioanalyzer (Agilent Technologies, Palo Alto, CA, USA) and a NanoDrop 2000 spectrophotometer (Thermo Fisher Scientific, Waltham, MA, USA), respectively. RNA-Seq was carried out by GeneDenovo Biotechnology Co. (Guangzhou, China) using an Illumina HiSeq TM 4000 instrument (Illumina, CA, USA).

For sequence data analysis, the clean reads of each RNA-Seq sample were mapped to the reference genome of *U. virens* using HISAT2 (v2.1.0). The TPM (Transcripts Per Kilobase Million) value was calculated to quantify gene expression abundance using RSEM software (version 0.6) [30]. The differentially expressed genes (DEGs) between two groups were analyzed using DESeq2 software (version 1.4.0). DEGs were determined with a *p*-value <0.05 and |log2foldchange| ≥1.5 [31,32]. All DEGs were mapped to the *U. virens* genome (PRJNA756324). The functional categories of DEGs were then subjected to enrichment analysis using the gene ontology (GO) (http://www.geneontology.org/, accessed on 26 June, 2023) and Kyoto Encyclopedia of Genes and Genomics (KEGG) (http://www.kegg.jp/, accessed on 26 June, 2023) databases.

### 2.7. qRT-PCR Assay

To validate the results of identified DEGs, real-time qRT-PCR analysis was performed on the DEGs related to vegetable growth and metabolism. The same RNA sample used for RNA-Seq was used for qRT-PCR validation. For detection of the expression of ustiloxin and ustilaginoidin biosynthesis-related genes, an independent assay collected the hyphae of *U. virens* grown on PSA plates treated with 10 ng/mL rapamycin or ethyl alcohol (as solvent control) for 14 d at 28 °C for qRT-PCR validation. The *U. virens* gene *α-tubulin* was used as an internal control. The corresponding primers are listed in Table S1. cDNA was synthesized from total RNA using the PrimeScriptTM RT Master Mix (TaKara, Dalian, China) and according to the manufacturer’s protocol. qRT-PCR analysis was performed using TB Green^R^ Premix Ex Taq^TM^ II (TaKara, Dalian, China) on an ABI QuantStudio 1 Plus (Applied Biosystems, Foster City, CA, USA). The relative gene expression levels were calculated using the 2^−ΔΔCT^ method. Each assay was repeated three times in technical replicates.

### 2.8. Statistical Analysis

All experiments were performed in at least three independent replicates. The data from each assessment were presented as mean ± standard deviation (SD). Data analysis was performed through one-way ANOVA followed by Duncan’s multiple-range test. *p*-values < 0.05 were considered significant.

## 3. Results

### 3.1. Molecular Components of the TOR Protein in U. virens

**Figure 1:**
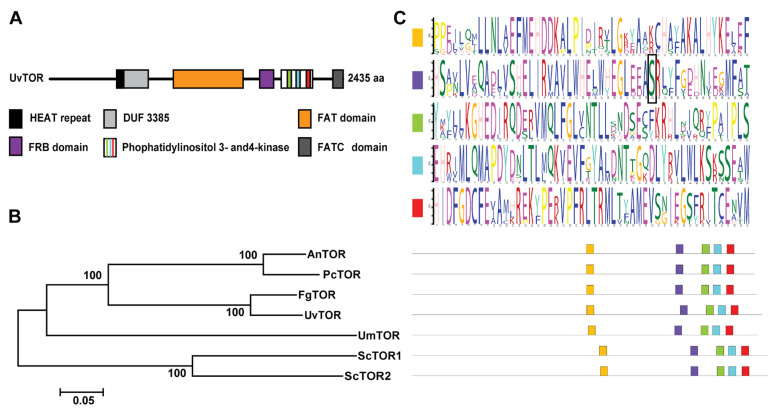
The kinase UvTOR in *U. virens* is evolutionarily conserved in fungal species. (**A**) The protein regions of UvTOR in *U. virens*. (**B**) Phylogenetic analyses of TOR proteins in *A. nidulans* (*An*), *P. chrysogenum* (*Pc*), *F. graminearum* (*Fg*), *U. virens* (*Uv*), *U. maydis* (*Um*) and *S. cerevisiae* (*Sc*). (**C**) Conserved catalytic motifs of TOR kinases in the indicated species. The conserved catalytic motifs in each TOR protein are shown in different color squares. The yellow color indicates the conserved catalytic motif of FAT domain. The purple color shows the conserved catalytic motif of FRB domain, And the green, blue and red colors indicate the three conserved catalytic motif located in Phophatidylinositol 3- and4-kinase domain. The black square marks the putative interaction site in TOR proteins and rapamycin receptor Fkbp12.

By analogy with the TOR genes in *S. cerevisiae*, we predicted that *UV8b_00876* encodes a TOR homologue gene (named *UvTOR*), located on the first chromosome of the *U. virens* (Table 1). Further analysis showed that the full-length genomic DNA of *UvTOR* spans about 7.3 kb, encoding a protein of 2435 amino acid residues (Figure 1A). Phylogenetic analyses revealed that UvTOR is evolutionarily conserved and highly homologous to TOR proteins in *A. nidulans*, *Penicillium chrysogenum*, *F. graminearum*, *U. virens*, *Ustilago maydis* and *S. cerevisiae*, especially showing a closer evolutionary relationship with FgTOR (Figure 1B). Differently from *S. cerevisiae*, there was only one TOR kinase in *U. virens* (Table 1). *ScKog1* and *ScLst8,* as the core components in TORC1, each showed one homologous gene by homologous comparison in the *U. virens* genome (Table 1). Alignment of UvTOR with TOR protein sequences from other species showed conserved catalytic motifs with significant identification (Figure 1C). Additionally, an evolutionarily conserved serine residue, reported to be involved in binding the rapamycin–FKBP12 complex, was found to be distributed in the FRB domain of TOR kinases (Figure 1C). These results indicate that the TOR signaling pathway is conserved in *U. virens*.

### 3.2. The TOR Signaling Pathway Is Involved in the Regulation of Hyphal Growth and Conidiation

**Figure 2:**
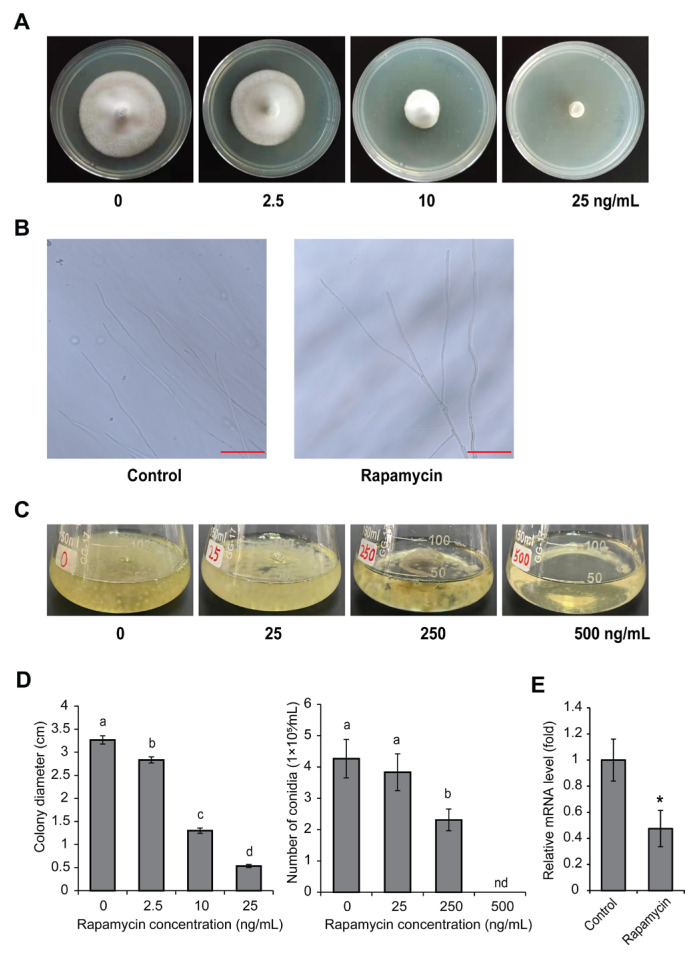
Effects of rapamycin on hyphal growth, hyphal branching, conidiation and expression level of *UvTOR* in *U. virens*. (**A**) Inhibitory effect upon vegetable growth of rapamycin in *U. virens.* The wild-type strain 57-2 was incubated for 14 d on potato sucrose agar (PSA) amended with concentrations of rapamycin from 0–25 ng/mL. (**B**) Hyphal morphology of *U. virens* grown on PSA with 10 ng/mL rapamycin. Scale bar represents 500 μm. (**C**) Inhibitory effect upon conidiation of rapamycin in *U. virens.* The wild-type strain was incubated for 14 d on potato sucrose agar (PSA) amended with concentrations of rapamycin from 0–25 ng/mL. Five mycelial plugs of stain 57-2 were inoculated in 50 mL PS liquid medium for 5 days supplemented with rapamycin at 0, 25, 250 or 500 ng/mL in a shaker with 150 rpm at 28 °C. (**D**) Colony diameters and conidia production under treatment of rapamycin. Representative data from three independent assays are shown as mean ± SD (*n* = 3). (**E**) Expression levels of *UvTOR* were verified by real-time qPCR. The *α-tubulin* of *U. virens* was used as an internal control. The results from three independent replicates are presented as mean ± SD (*n* = 3). Different lowercase letters and an asterisk show statistically significant differences according to one-way analysis of variance followed by Duncan’s multiple range test (*p <* 0.05).

To elucidate the roles of the TOR signaling pathway in fungal development, the sensitivity to the TOR kinase inhibitor rapamycin of *U. virens* was assayed after treatment with a series of concentrations of rapamycin from 0–25 ng/mL. The cultivation of *U. virens* on PSA plates amended with rapamycin at concentrations of 10 ng/mL led to a ~60% reduced colony size and the radical growth of *U. virens* was nearly completely inhibited on PSA at higher concentrations (25 ng/mL) (Figure 2A). Microscopic observation showed that hyphae treated with rapamycin at 10 ng/mL had more branches compared with the control (Figure 2B). Furthermore, after culturing in liquid PS medium for 5 days, fewer conidia were produced after treatment with different concentrations of rapamycin (Figure 2C). Conidia number statistics showed that there was no significantly difference in spore production between the treatment of 25 ng/mL and control, while conidia production was dramatically decreased under 250 ng/mL of rapamycin (Figure 2D). Spores were not observed in PS medium with concentrations of rapamycin above 500 ng/mL (Figure 2C,D). We next confirmed that the expression of *UvTOR* was significantly reduced after treatment with rapamycin at 10 ng/mL (Figure 2E), which was consistent with the limit of hyphae growth and conidiation upon rapamycin treatment. Collectively, the observed effects of TOR kinase inhibitors suggest that the TOR signaling pathway plays important roles in the vegetative growth and conidiation of *U. virens.*

### 3.3. The TOR Signaling Pathway Is Associated with Pathogenicity

**Figure 3:**
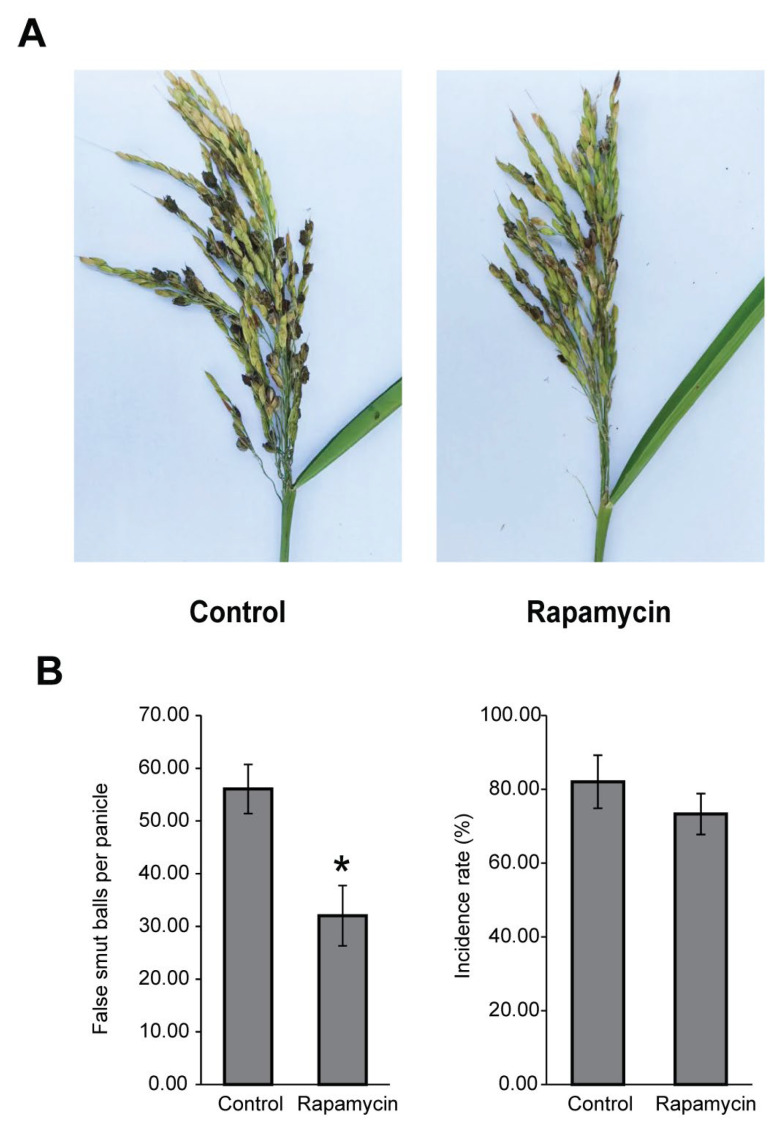
TOR inhibition reduces the virulence of *U. virens*. (**A**) Infected phenotype of rice panicles at 6 weeks post-inoculation. Rice panicles were inoculated with a spore suspension from the wild type (57-2) and a spore suspension mixed with 10 ng/mL rapamycin. (**B**) The number of false smut balls per panicle and incidence was measured. Line bars denote the standard errors of three experiments. The asterisk showed statistically significant differences according to one-way analysis of variance followed by Duncan’s multiple range test *(p <* 0.05).

Given the decreased hypha growth rate and conidial production after being treated with rapamycin, we speculate that the TOR signaling pathway regulates *U. virens* pathogenicity. The panicles of susceptible rice cultivar CY919 were injected with a *U. virens* conidial suspension mixed with rapamycin (10 ng/mL). After 6 weeks of incubation, fewer false smut balls were observed on inoculated panicles when infected with *U. virens* under rapamycin treatment compared with that of solvent treatment (Figure 3A). The number of false smut balls per panicle was significantly decreased to 32.03 ± 5.72 after inoculated with a *U. virens* spore suspension mixed with 10 ng/mL rapamycin (Figure 3B). Although the incidence rate of the rapamycin treatment group was reduced to 73.30% ± 5.58%, there was no significant difference in the incidence rate between the rapamycin treatment group and the solvent control (Figure 3B). These data suggested that the TOR signaling pathway is critical for the virulence of *U. virens*.

### 3.4. The TOR Signaling Pathway Regulates Related Gene Expression to Control Growth and Metabolism

**Figure 4:**
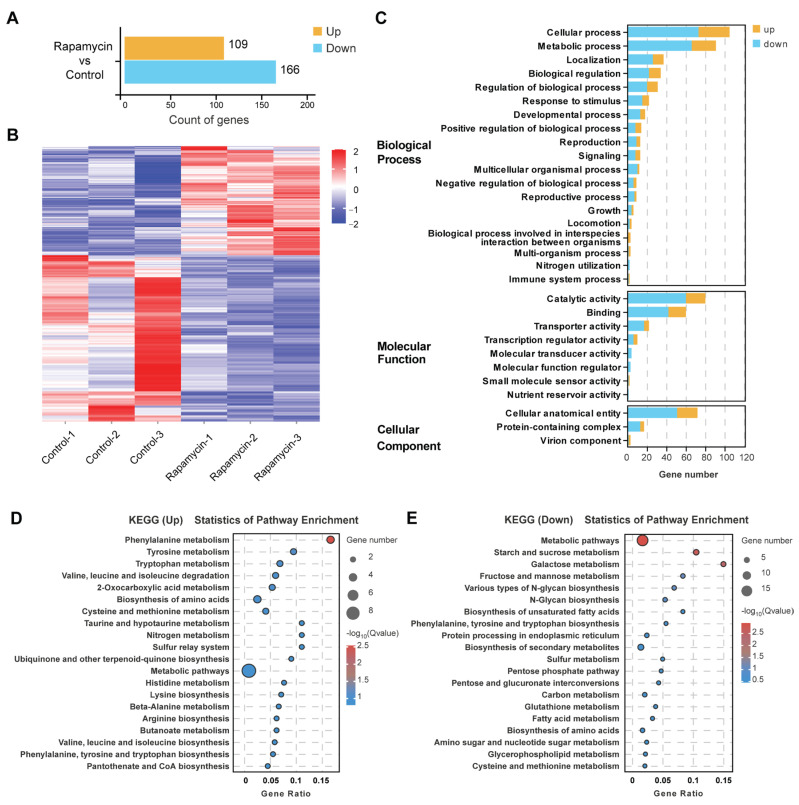
RNA profiling of *U. virens* in response to rapamycin. (**A**) The number of differentially expressed genes (DEGs). (**B**) Expression pattern of DEGs. Red in the heat map denotes up-regulation while blue denotes down-regulation. (**C**) The top 30 enriched GO terms. (**D**) The top-20 enriched KEGG pathways for up-regulated DEGs. (**E**) The top 20 enriched KEGG pathways for down-regulated DEGs.

The growth inhibition and virulence assay showed that treatment with 10 ng/mL rapamycin lead a significantly reduced colony size and pathogenicity of *U. virens*, which indicated the transcription level of genes associated with cell growth and metabolism had changed. To further investigate the role of the TOR signaling pathway in *U. virens*, we compared the transcriptome profile of the WT strain with that of rapamycin-treated WT strains through RNA sequencing (RNA-Seq). Upon rapamycin treatment, there was a total of 275 differentially expressed genes (DEGs), consisting of 109 up-regulated DEGs and 166 down-regulated DEGs, in a statistically significant manner (*p*-value < 0.05 and |log2foldchange| ≥ 1.5) (Figure 4A,B and Tables S1 and S2). Enrichment analysis of gene ontology (GO) terms showed that genes involved in the rapamycin response of the wild type were mainly related to cellular process, metabolic process, localization, biological regulation, catalytic activity, protein binding and cellular anatomical entity, which indicated the global function of the TOR signaling pathway in the regulation of growth and secondary metabolism in *U. virens* (Figure 4C and Table S3). Furthermore, in the top 20 terms of GO enrichment in the Cellular Component, most DEGs were associated with RNA processing and DNA replication and reparation (Figure S2 and Table S3), which is in agreement with the conserved function of TOR in the regulation of ribosome biogenesis, protein synthesis and transcription. To gain insight into the key biological pathways that responded to rapamycin treatment, the Kyoto Encyclopedia of Genes and Genomes (KEGG) enrichment was analyzed (Figure 4D,E and Table S4). In the up-regulated top most enriched pathways, most enrichments participated in amino acid and acetyl-CoA metabolism, such as ‘tyrosine metabolism’, ‘valine, leucine and isoleucine degradation’ and ‘pantothenate and CoA biosynthesis’, all of which may in turn promote the synthesis of polyketide and ribosomal peptides (Figure 4D). In the top 20 most enriched down-regulated pathways, the majority were involved in carbohydrate and fatty acid metabolism and may inhibit cell growth through energy synthesis and nutrient absorption (Figure 4E). Subsequently, RT-qPCR was performed to validate the accuracy of the RNA-Seq data, for which the results were generally consistent with the expression pattern from the RNA-Seq data (Figure S1). The RT-qPCR results showed that the amino acid and acetyl-CoA metabolism genes *UV8b_01035*, *UV8b_01424*, *UV8b_03324* and *UV8b_07650* were significantly up-regulated upon rapamycin treatment and the expression level of the carbohydrate metabolism-related genes *UV8b_00190*, *UV8b_04708*, *UV8b_04720*, *UV8b_05883*, *UV8b_06058*, *UV8b_07614*, *UV8b_03450* and *UV8b_04707*, as well as the fatty acid metabolism-related genes *UV8b_01293*, *UV8b_06209*, *UV8b_07613* and *UV8b_01512*, were reduced after being treated with rapamycin. These results were generally consistent with the transcriptome data. Altogether, these results indicated the conserved TOR function in the regulation of growth and metabolism in *U. virens.*

### 3.5. Rapamycin Exhibits a Strong Induction of Mycotoxin Biosynthesis

**Figure 5:**
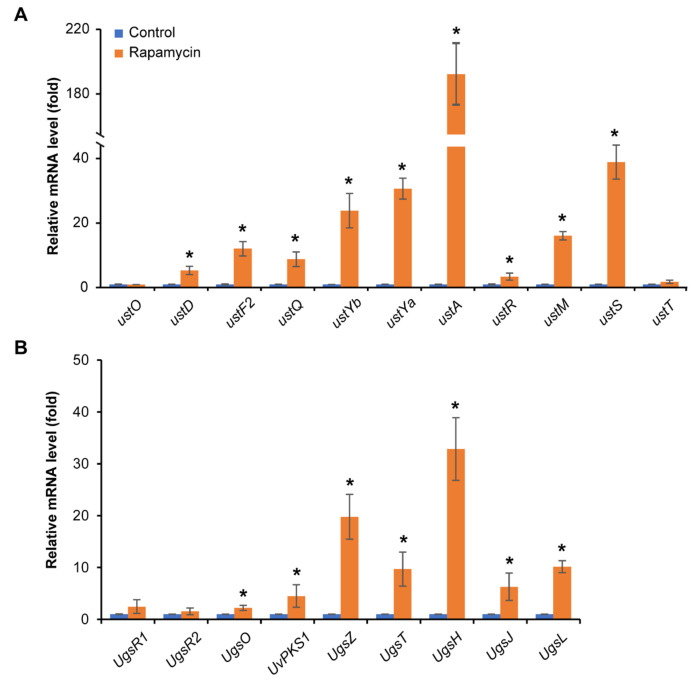
Rapamycin treatment induces the expression of mycotoxin biosynthesis-related genes in *U. virens*. (**A**) The expression of ustiloxin biosynthesis-related genes, including *ustO, ustD, ustF2, ustQ, ustYb, ustYa, ustA, ustR, ustM, ustS and ustT*, was confirmed by real-time qPCR. (**B**) The expression of ustilaginoidin biosynthesis-related genes, including *ugsR1, ugsR2, ugsO, UvPKS1, ugsZ, ugsT, ugsH, ugsJ and ugsL*, was detected through real-time qPCR. The *α-tubulin* of *U. virens* was used as an internal control. The results from three independent replicates are presented as mean ± SD (*n* = 3). The asterisks show statistically significant differences according to one-way analysis of variance followed by Duncan’s multiple range test (*p <* 0.05).

Based on the up-regulated expression of amino acid and acetyl-CoA metabolism through KEGG enrichment analysis, we hypothesize that the treatment of rapamycin led to the promotion of the synthesis of related secondary metabolites, especially mycotoxins. The expression of ustiloxin and ustilaginoidin biosynthesis-related genes was detected by real-time qPCR analysis. Interestingly, the ustiloxin biosynthesis-related genes *ustD* (*UV8b_06021*), *ustF2* (*UV8b_06023*), *ustQ* (*UV8b_06024*), *ustYb* (*UV8b_06025*), *ustYa* (*UV8b_06026*), *ustA* (*UV8b_06027*), *ustR* (*UV8b_06028*), *ustM* (*UV8b_06029*) and *ustS* (*UV8b_06030*) [6,7] and the ustilaginoidin biosynthesis-related genes *ugsO* (*UV8b_01131*), *UvPKS1* (*UV8b_01134*), *ugsZ* (*UV8b_01135*), *ugsT* (*UV8b_01136*), *ugsH* (*UV8b_01137*), *ugsJ* (*UV8b_01138*) and *UV8b_01139* (*ugsL*) [4] were significantly up-regulated after rapamycin treatment (Figure 5). The expression of the key biosynthesis gene *ustA,* which is responsible for the precursor synthesis of ustiloxins, was increased more than 190-fold (Figure 5A). In addition, we quantified the level of ustilaginoidin generation in the mycelia collected from the *U. virens* strain grown on PSA plates amended with 10 ng/mL rapamycin by HPLC. The HPLC assay demonstrated that more ustilaginoidins were produced after the rapamycin treatment than for the control (Figure S3). These results revealed that the TOR signaling pathway regulates the generation of ustilaginoidins and the expression of ustiloxin biosynthesis genes.

## 4. Discussion

*Ustilaginoidea virens* infects and colonizes rice florets that further form false smut balls, which causes significant reductions in rice yield and generates multiple types of mycotoxins. Although the TOR signaling pathway has been reported to play critical roles in regulating vegetative growth and metabolism in fungi, there is a lack of knowledge about the TOR signaling pathway in virulence and mycotoxin biosynthesis in *U. virens*. In this study, we demonstrate for the first time that *U. virens* possesses a functional TOR signaling pathway that positively governs vegetative growth, conidiation and pathogenicity, but negatively regulates the biosynthesis of mycotoxins.

The TOR signaling pathway is essential for controlling cell growth and secondary metabolism in modulating downstream biological processes [33,34]. Previous studies reported that rapamycin strongly inhibits hyphal growth and leads to the generation of more hyphal branches of *F. graminearum* and *B*. *cinerea* [18,25]. In this study, similar results were observed in *U. virens* after treatment with rapamycin (Figure 2A,B), suggesting that TOR is a key regulator involved in cell growth. RNA-Seq analysis of *U. virens* after treatment with rapamycin showed that the down-regulated DEGs were enriched in carbohydrate- and fatty acid-related metabolism pathways (Figure 4E and Figure S1 and Table S4), which supported the result of the growth inhibition assay of *U. virens* upon rapamycin treatment. Many studies have shown that carbohydrate and fatty acid metabolism play an important role in growth and are closely related to pathogenicity [35]. *M. oryzae* loses the abilities of infection and growth after deleting the *TPS* gene, which breaks the starch and sucrose metabolic pathway [36]. Metabolites involved in carbohydrate metabolism were significantly accumulated in a strongly virulent *U. virens* strain, PXD25, compared with the weakly virulent strain GY900 after infection, which indicated that carbohydrate metabolism plays an essential role in the virulence of *U. virens* [37]. Further, fatty acid metabolism is critical for maintaining fatty acid homeostasis, which involves the production of the cell membrane and energy to sustain cell life [38,39]. *M. oryzae* with dysfunctional fatty acid metabolism exhibits defects in cell growth, perosome biogenesis and appressorium development, all of which then attenuated *M. oryzae* virulence [40]. The biochemical inhibition of TOR kinase assay combined with transcriptome analysis revealed that the TOR pathway controls cell growth through regulating the carbohydrate- and fatty acid-related metabolism of *U. virens*. In addition, similar to previous reports in *F. graminearum* [18] and *B*. *cinerea* [25], rapamycin treatment also led to a severe reduction of conidiation in *U. virens* at concentrations of more than 250 ng/mL (Figure 2C,D), suggesting that it is possible to restrict the spread of *U. virens* by inhibiting the function of the TOR signaling pathway. TOR inhibition reduces pathogenicity in *F. graminearum*, *B*. *cinerea* and *M. oryzae* and is associated with losing the abilities of hyphal growth, conidiation or appressorium development [18,20,25]. Consistent with these reports, we also observed a reduction of pathogenicity in *U. virens* under rapamycin treatment. Taken together, these findings suggest that the TOR signaling pathway regulates infection and host colonization by controlling phytopathogen growth and proliferation in *U. virens*.

Filamentous fungi produce an enormous amount of secondary metabolites, and the TOR signaling pathway acts as a master regulator to control secondary metabolism, especially mycotoxins. Inhibition of TOR function reduced gibberellin and bikaverin biosynthesis through affecting the expression of *AreA*-controlled secondary metabolite genes in *F. fujikuroi* [41]. Exogenous rapamycin treatment affected the biosynthesis of terpenoids and polyketones in *Trichoderma atroviride* by seriously inhibiting the activities of various downstream hydrolases [42]. Contrary to previous research, we observed that the TOR signaling pathway negatively regulated the production of ustiloxins and ustilaginoidin in *U. virens*. This means that the TOR signaling pathway in *U. virens* showed a different regulation pattern in secondary metabolism. A previous study in *U. virens* showed that the accumulation of amino acids or acetyl-CoA promotes the synthesis of polyketide and ribosomal peptides [27]. The ustilaginoidin biosynthesis begins with the condensation of one acetyl-CoA and six malonyl-CoA molecules by UvPKS1 [4], and ustA contains a five-fold repeat of short peptides, including “Tyr-Val-Ile-Gly (YVIG)”, which further translate and perform the core peptide of ustiloxins [6,7]. The RNA-Seq data showed that amino acid- and acetyl-CoA-related metabolism were up-regulated upon rapamycin treatment (Figure 4D and Figure S2). We speculated that the variation of intracellular acetyl-CoA and amino acid levels will influence ustilaginoidin and ustiloxin generation. The up-regulated expression of genes related to ustilaginoidin and ustiloxin synthesis was consistent with our speculation. The expression of genes related to ustilaginoidin synthesis was increased ranging from 1 to 30 times (Figure 5B). The HPLC assay demonstrated that more ustilaginoidins were produced after rapamycin treatment than in the control (Figure S3). Further, the nine tested genes in the ustiloxin biosynthesis cluster were transcriptionally up-regulated upon rapamycin treatment. In particular, the expression of the ustiloxin precursor synthetic gene *ustA* increased more than 190 times. Unfortunately, we failed to detect ustiloxins in the mycelia collected from *U. virens* grown on PSA plates amended with rapamycin. A previous study showed that ustiloxins were only isolated and detected from rice false smut balls [1,5]. We speculated that there are other regulatory factors during the formation of rice false smut balls that induce ustiloxin production. The laboratory culture situation for *U. virens* provides a single-nutrient environment that cannot stimulate ustiloxin biosynthesis. Future research may attempt to use host-induced gene silencing of UvTOR in *U. virens* and detect the level of ustiloxins in rice false smut balls. Up to now, there has been a lack of relevant research on the regulatory mechanisms of ustiloxins that are threatening to human and animal food safety [43]. Our study has indicated the TOR signaling pathway is a key regulator of mycotoxin biosynthesis, especially for ustiloxins, in *U. virens*. Therefore, further investigation is needed to determine the critical genes downstream of the TOR signaling pathway involved in regulating ustiloxin synthesis in *U. virens*.

To our knowledge, this is the first comprehensive study of the TOR signaling pathway in *U. virens.* Biochemical inhibition of TOR kinase revealed that the TOR signaling pathway plays a significant role in pathogenesis, probably through positively regulating vegetative growth and conidiation, whereas it negatively modulated ustiloxin production by inhibiting the gene expression of mycotoxin biosynthesis. However, we failed to obtain a gene deletion mutant for *UvTOR* after screening more than 200 transformants, suggesting that *UvTOR* may be essential for cell life in *U. virens*, which is consistent with the previous studies of *F. graminearum*, *B*. *cinerea* and *M. oryzae* [18,20,25]. Transcriptomic analysis showed that the up-regulated DEGs were enriched in the amino acid- and acetyl-CoA-related metabolism pathways and the down-regulated DEGs were enriched in the carbohydrate- and fatty acid-related metabolism pathways. The results are helpful in revealing that the TOR signaling pathway acts as a central regulator involved in numerous of biological and physiological processes in *U. virens*. In summary, our results deepen the understanding of the mechanisms behind the TOR signaling pathway’s regulation of pathogenesis and mycotoxin biosynthesis in *U. virens*, suggesting that *UvTOR* could be a potential and promising candidate target for controlling rice false smut.

## Figures and Tables

**Table 1:** The *U. virens* genes homologous to the TOR complex genes from *Saccharomyces cerevisiae*.

	Name	Gene ID in *U. virens*	Homologue in *S. cerevisiae*	Blast E Value
TORC1	*UvTOR*	UV8b_00876	*ScTor1*	0
	*UvKog1*	UV8b_04344	*ScKog1*	4 × 10^−134^
	*UvLst8*	UV8b_04658	*ScLst8*	1 × 10^−153^
	*-*	-	*ScTco89*	-
TORC2	*-*	-	*ScTor2*	-
	*UvAvo1*	UV8b_06451	*ScAvo1*	1 × 10^−18^
	-	-	*ScAvo2*	-
	-	-	*ScAvo* *3*	-
	-	-	*ScBit61*	-
	-	-	*Sc* *Bit2*	-

## Data Availability

All data presented in the study are included within the article and its Supplementary Files or have been deposited in NCBI with accession PRJNA1231200.

## Supplementary Materials

The following supporting information can be downloaded at: https://www.mdpi.com/article/10.3390/jof11040239/s1. Figure S1: Validation of RNA-Seq data by qRT-PCR. Figure S2: Top 20 terms of GO enrichment in Cellular Component. Figure S3: HPLC assay to quantify ustilaginoidin production of *U. virens* grown on PSA plates amended with 10 ng/mL rapamycin. Table S1: All genes detected between rapamycin treatment and control. Table S2: DEGs between rapamycin treatment and control. Table S3: Enriched GO terms. Table S4: Enriched KEGG pathway. Table S5: Primers used in the qRT-PCR assay for validation of RNA-Seq data.
