# Supplementary figures and images for: The TOR Signaling Pathway Governs Fungal Development, Virulence and Ustiloxin Biosynthesis in Ustilaginoidea virens
Source: J Fungi (Basel). 2025 Mar 21;11(4):239. doi: 10.3390/jof11040239 (PMC12028740; doi:10.3390/jof11040239)

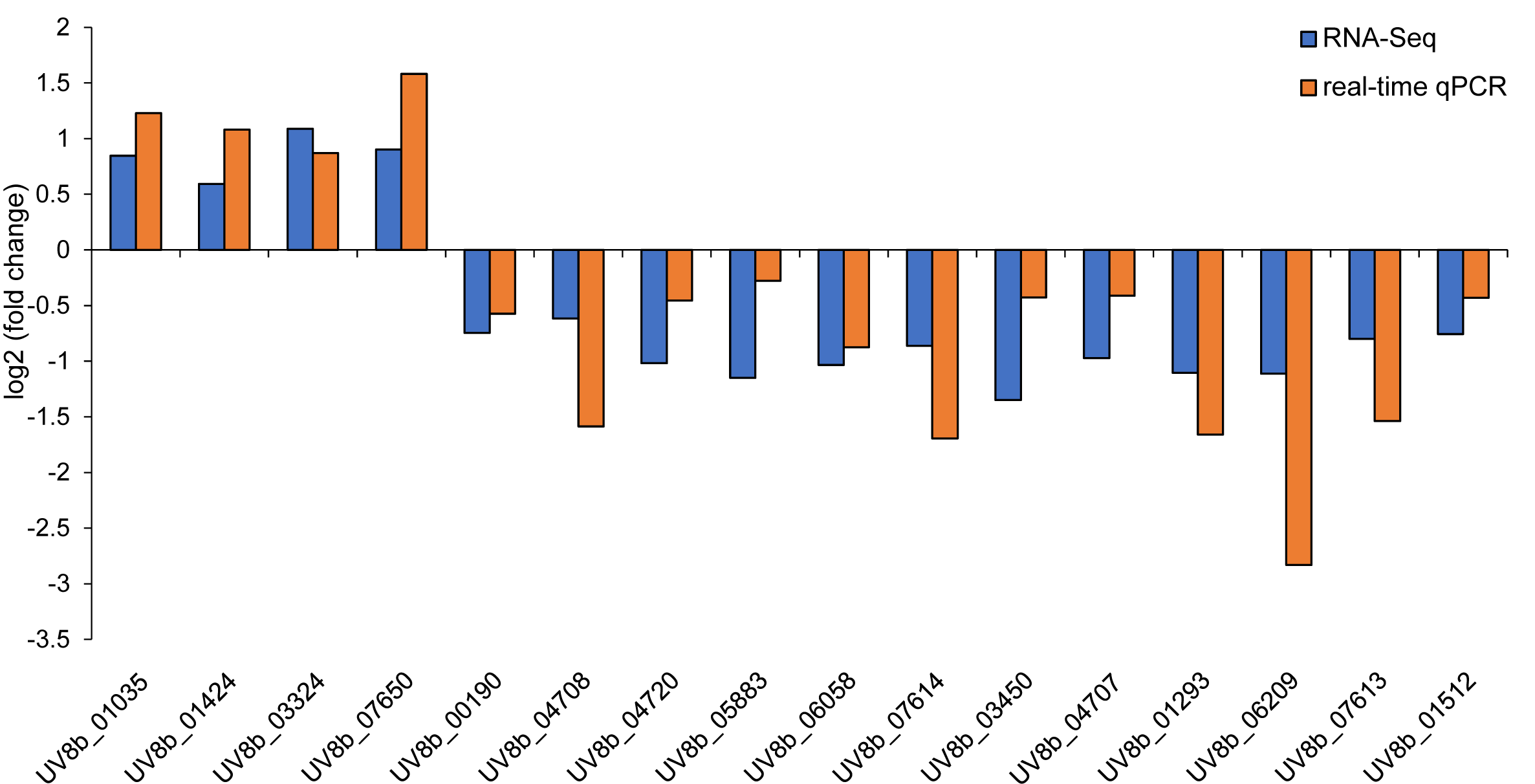

Supplement: Supplementary file 1 [file jof-11-00239-s001.zip › Figure S1.tif]

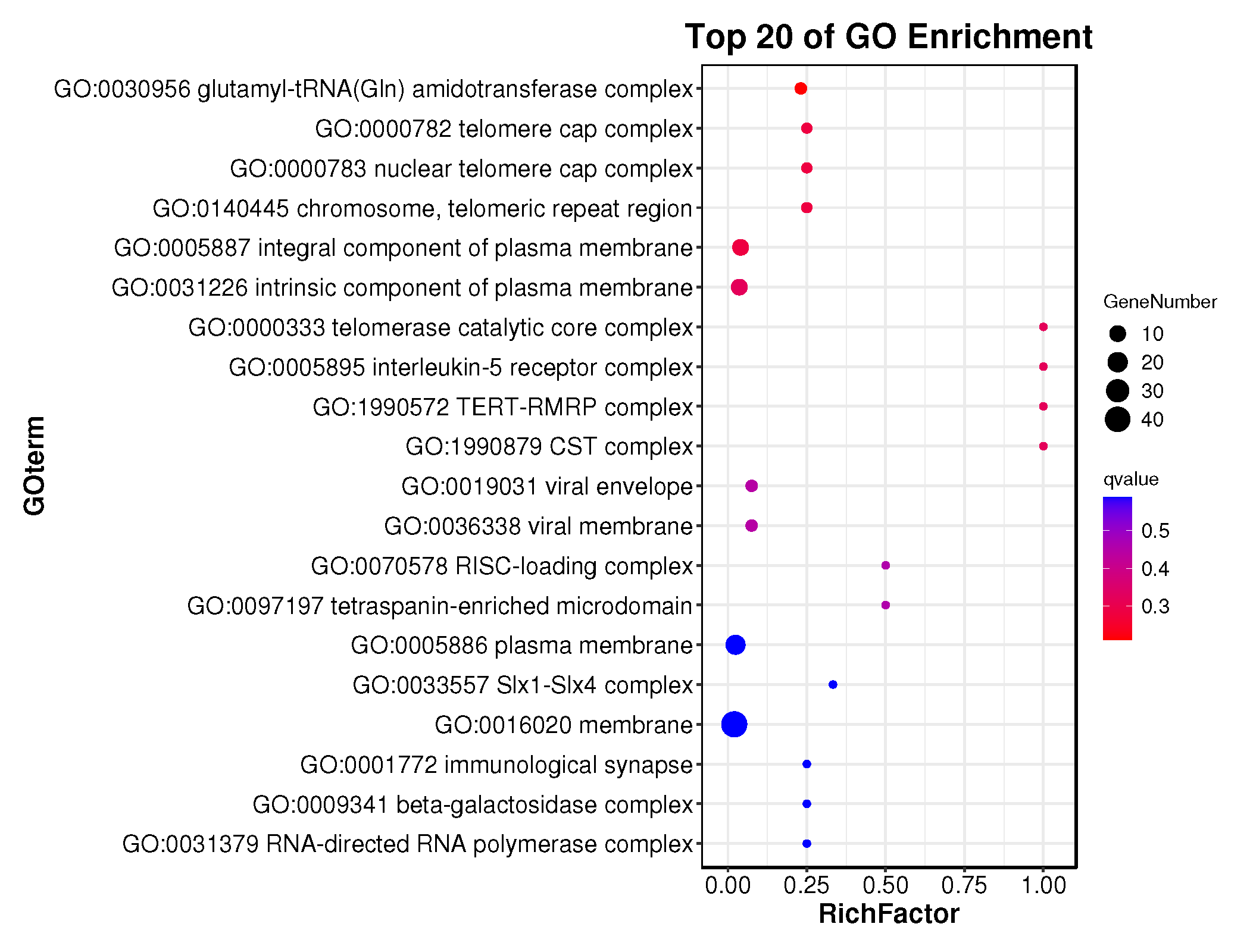

Supplement: Supplementary file 1 [file jof-11-00239-s001.zip › Figure S2.tiff]

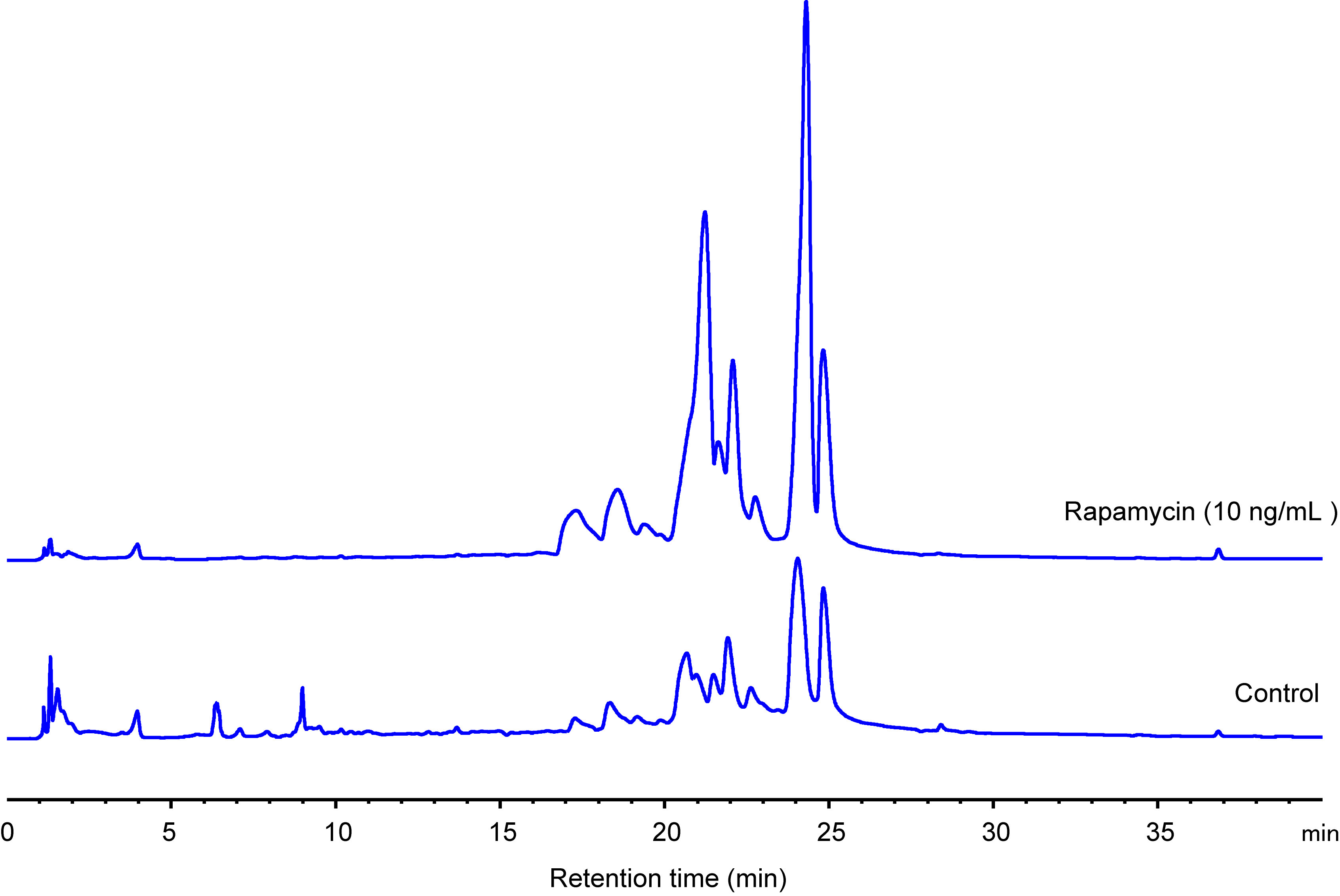

Supplement: Supplementary file 1 [file jof-11-00239-s001.zip › Figure S3.tif]
